# Repeated mosquito net distributions, improved treatment, and trends in malaria cases in sentinel health facilities in Papua New Guinea

**DOI:** 10.1186/s12936-019-2993-6

**Published:** 2019-11-12

**Authors:** Daniela Rodriguez-Rodriguez, Seri Maraga, Lina Lorry, Leanne J. Robinson, Peter M. Siba, Ivo Mueller, Justin Pulford, Amanda Ross, Manuel W. Hetzel

**Affiliations:** 1 Swiss Tropical and Public Health Institute, Basel, Switzerland; 2 University of Basel, Basel, Switzerland; 3 Papua New Guinea Institute of Medical Research, Goroka and Madang, Papua New Guinea; 4 Walter and Eliza Hall Institute of Medical Research, Melbourne, Australia; 5 Burnet Institute, Melbourne, Australia; 6 Institut Pasteur, Paris, France; 7 Liverpool School of Tropical Medicine, Liverpool, UK

**Keywords:** Malaria, Incidence, Vector control, Artemisinin-based combination therapy, *Plasmodium falciparum*, *Plasmodium vivax*

## Abstract

**Background:**

Long-lasting insecticidal nets (LLIN), improved diagnosis and artemisinin-based combination therapy (ACT) have reduced malaria prevalence in Papua New Guinea since 2008. Yet, national incidence trends are inconclusive due to confounding effects of the scale-up of rapid diagnostic tests, and inconsistencies in routine reporting.

**Methods:**

Malaria trends and their association with LLIN and ACT roll-out between 2010 and 2014 in seven sentinel health facilities were analysed. The analysis included 35,329 fever patients. Intervention effects were estimated using regression models.

**Results:**

Malaria incidence initially ranged from 20 to 115/1000 population; subsequent trends varied by site. Overall, LLIN distributions had a cumulative effect, reducing the number of malaria cases with each round (incidence rate ratio ranging from 0.12 to 0.53 in five sites). No significant reduction was associated with ACT introduction. *Plasmodium falciparum* remained the dominant parasite in all sentinel health facilities. Resurgence occurred in one site in which a shift to early and outdoor biting of anophelines had previously been documented.

**Conclusions:**

LLINs, but not ACT, were associated with reductions of malaria cases in a range of settings, but sustainability of the gains appear to depend on local factors. Malaria programmes covering diverse transmission settings such as Papua New Guinea must consider local heterogeneity when choosing interventions and ensure continuous monitoring of trends.

## Background

Malaria in Papua New Guinea (PNG) was described by Koch in 1900 [1, 2] and to date malaria transmission remains endemic in PNG especially in areas below 1400 m altitude [3, 4]. Over the last century, the epidemiology of malaria in PNG has been influenced by control and elimination efforts [2, 4–6]. In the 1950s to 1980s, PNG had joined efforts of the Global Malaria Eradication Programme with spraying of dichlorodiphenyltrichloroethane (DDT) and mass drug administration (primarily chloroquine) [6]. The programme concluded before elimination was achieved and malaria resurged in the 1990s [2, 4]. In 2004, control efforts were re-intensified with funding from the Global Fund to Fight AIDS, Tuberculosis and Malaria to the PNG national malaria control programme (NMCP). Since then, the NMCP has promoted: (1) the country-wide free distribution of long-lasting insecticidal nets (LLIN); (2) behaviour change campaigns; and, (3) the scaling-up of parasitological diagnosis by rapid diagnostic test (RDT) or microscopy, together with the introduction of artemisinin-based combination therapy (ACT), specifically artemether-lumefantrine [7].

Since this last scale-up, the malaria burden in PNG has steadily decreased as reflected in declining prevalence of infection [8], incidence [9] and transmission [10]. However, malaria control efforts are intrinsically affected by the great environmental and socio-cultural diversity across the country, including a major mountain range over the length of the main island, dense rainforests in the highlands, lowland and coastal areas, and large wetlands surrounding major rivers [2]. This diversity has influenced human population distribution, human behaviour and mosquito ecology. All this, and the presence of four human pathogenic malaria parasites (*Plasmodium falciparum, Plasmodium vivax, Plasmodium malariae, Plasmodium ovale*), results in a complex malaria epidemiology with heterogeneous levels of endemicity [2, 3, 5, 11].

While changes in malaria prevalence have been consistently investigated since 2008 [8], national trends in malaria incidence are inconclusive and difficult to interpret due to confounding effects of the scale-up of RDTs, changes in health facility reporting forms, and inconsistencies in routine reporting [7].

This study aimed to estimate malaria trends over time (2010–2014) in seven sentinel health facilities (SHF) and assess the effect of repeated household-level distributions of LLINs and the introduction of ACT in distinct epidemiological settings across PNG.

## Methods

### Study design

**Fig. 1:**
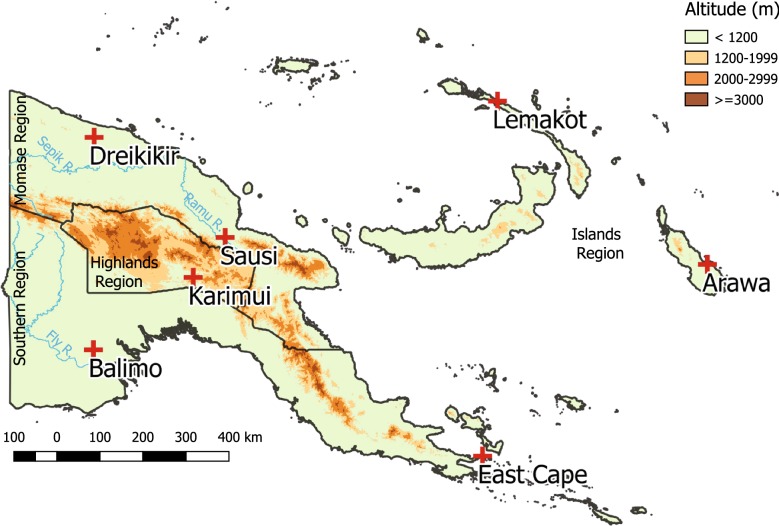
Location of sentinel health facilities in Papua New Guinea (red crosses). Dark lines indicate regional boundaries

A health facility based longitudinal study established surveillance of malaria cases, severity of symptoms, net use and parasite species composition in seven purposively selected sentinel health facilities from 2010 to 2014 (Fig. 1). Intervention roll-out was recorded for each site. In four sites, a baseline population census was conducted in the catchment areas of the SHF. In addition, satellite data was extracted for each site and for the duration of the surveillance period to complement clinical data with environmental data.

### Study sites

SHFs were functioning health centres and one sub-centre (Sausi), accessible by road or air, with a catchment population of at least 5000 people, that regularly reported malaria cases. The catchment area defined by the local authorities was adopted for the surveillance. Surveillance activities were established as part of the continuous independent evaluation of the NMCP [4, 7]. Seven SHFs were selected; two each from Southern, Momase and Islands regions, and one from the Highlands region where the malaria burden is lower due to higher altitudes [3]. A description of each site is provided in Additional file 1.

### Data collection

#### Clinical data at the sentinel health facilities

The surveillance period in each SHF and the timing of LLIN distribution rounds and introduction of ACT as first-line treatment are provided in Additional file 2.

All outpatient cases attending a SHF were routinely screened for a history of self- reported fever in the previous 3 days (‘fever case’). Study-related procedures were performed by registered nursing officers or community health workers (“study nurses”) trained in the proper performance of capillary blood sample collection, the use and reading of RDT test kits according to the manufacturer’s guidelines, the disposal of bio-hazardous waste, and the recording or results according to the study protocol. The study nurses were based full-time at the facility and collected a capillary blood sample by finger-prick from all consenting fever patients for: (1) point-of-care diagnosis of malaria by RDT; (2) thick and thin blood smear for malaria diagnosis by light microscopy; and, (3) measurement of haemoglobin (Hb) concentration. All RDT-positive cases were considered ‘malaria cases’ in this analysis. Severe malaria was defined as RDT-positive cases presenting with at least one of the following danger signs: impaired consciousness (including coma or convulsions), difficulty breathing, or severe anaemia (Hb < 8 g/dl, or < 7 g/dl for children under 5 years old and pregnant women). Demographic details of the patients (age, sex, pregnancy status) and self-reported mosquito net use the previous night were recorded on paper case report forms alongside selected clinical indicators, including axillary temperature, Hb measurement and RDT results. Patients were then transferred to a health facility clinician for further examination. The final diagnosis as determined by the health facility clinician and prescribed treatment were recorded in the same case report form. The study team ensured availability of RDTs during the surveillance period.

RDTs (ICT Malaria Combo HRP2/aldolase, ICT Diagnostics, South Africa) were used following manufacturer’s guidelines. A sub-sample was further examined by light microscopy at the Papua New Guinea Institute of Medical Research (PNGIMR) to identify the *Plasmodium* species (Additional file 3). Microscopy slides were fixed with methanol (thin smear), stained with Giemsa (thin and thick smear) and read independently by two microscopists. Discordant reads were confirmed with a third read by a senior microscopist (World Health Organization level 1 or 2). The number of parasites was counted for 200 white blood cells and a slide was declared negative after reading a minimum of 200 thick film fields. Hb concentration was measured using a HemoCue Hb 201+ Analyser (HemoCue AB, Sweden) and axillary temperature with a digital thermometer.

#### Demographic composition

A population census was conducted in the catchment areas of East Cape, Karimui, Sausi and Lemakot at the beginning of the surveillance period. The available funding was insufficient to conduct a baseline census in the three remaining SHFs. For the census village leaders assisted in the identification of households and enumeration of household members. The variables captured for each household included: household size, and age and sex of each household member. The annual population growth rate of 3.1% was obtained from the National Statistics Office [12].

#### Environmental data

Rainfall (product 3B43) and Enhanced Vegetation Index (EVI; products MOD13Q1 and MOD13A3) data were extracted from remote-sensing databases by the Tropical Rainfall Measuring Mission (TRMM) and the Earth Observing System (EOS) respectively. Rainfall data were accessed using the Mirador system in the NASA Goddard Earth Sciences Data and Information Services Center (GES DISC) website [13–15]. EVI data were accessed using the NASA Earth Data Search website [16]. In addition the annual occurrence of the El Niño/La Niña phenomena was extracted from the NASA Earth Observatory [17]. Key environmental variables are available in Additional file 4.

### Data analysis

Statistical data analysis was conducted using Stata/IC v.13.1 (Stata Corp LP., College Station, USA). Monthly data were graphically displayed to visualize trends in the numbers of fevers and malaria cases by SHF. Monthly accumulated rainfall in mm (product 3B43), was included as proxy of site-specific seasonality [13–15]. Missing periods in surveillance data reflect temporary unavailability of study nurses due to leave or staff change and were not related to particular times of the year.

The annual proportions of RDT-positive fever cases (RDT positivity) were calculated with 95% exact confidence interval (CI). The *Plasmodium* species composition was estimated from the light microscopy results. Details are provided in Additional file 3.

For the four sites with available census data, malaria incidence (all cases with a positive RDT) and ‘severe malaria’ incidence were calculated per 1000 population per year. Population denominators were adjusted for an annual growth rate of 3.1%.

The association between the roll-out of interventions (each of the three rounds of LLIN distribution and of the introduction of ACT as first-line treatment for test-confirmed malaria) and the number of malaria cases was assessed using regression models. The number of cases was used as the outcome since denominators were only available in four sites. To investigate the effect of LLIN distribution rounds, malaria cases were disaggregated by age groups for each LLIN distribution round. Regression models were used to assess the effect of both interventions simultaneously in the seven sites. The LLIN distribution variable had three different values, one for each period between LLIN distributions. The ACT variable was binary, with value zero before the introduction of ACT and value one thereafter. In preliminary analyses, time since the intervention was included as a variable. However, due to the limited number of observations for each site and the need for simplicity for interpretation these variables were not included in the final model.

Negative binomial regression was used to estimate the effect of the interventions on the monthly aggregate number of malaria cases. Fixed effects were included for the interventions and further covariates. Separate models were applied for each SHF after first establishing that the effects of the LLIN rounds were significantly different between sites using interactions terms. Due to convergence limitations, Poisson regression was used for the model with interactions.

The use of environmental variables such as rainfall (with and without time-lag) and EVI was explored. Initially rainfall was included in the model as a monthly mean per day and alternatively as accumulated monthly aggregate. Introduction of these variables in the model was explored with and without time-lags (1 month and 2 months). EVI variables were similarly explored in the model. The monthly averages of two different EVI products were introduced in the model with and without time-lag. These variables were later omitted from the model due to poor predictive ability. Finally, the estimates were adjusted for El Niño and La Niña annual occurrence. El Niño/La Niña variable was introduced in the model as a categorical variable with 3 possible values for annual occurrence (El Niño in 2010, La Niña in 2011 and 2012 and none in 2013 and 2014).

## Results

During the surveillance period, a total of 35,329 fever cases were recorded across all SHFs. RDT results were available for 98% (range: 94–99%) of all cases (Table 1). The pooled RDT positivity was 32%. Site-specific RDT positivity ranged from 4% in Balimo to 49% in East Cape.

**Table 1:** Number of fever cases and rapid diagnostic test result by sentinel health facility

Health facility	Fever cases N	RDT positive N (%, 95% CI)	RDT not done N (%, 95% CI)
Balimo	1596	57 (4, 3–5)	100 (6, 5–8)
East Cape	7311	3601 (49, 48–50)	137 (2, 1.6–2.2)
Karimui	3166	583 (18, 17–20)	34 (1, 0.7–1.5)
Dreikikir	3869	876 (23, 21–24)	27 (0.7, 0.5–1.0)
Sausi	6450	1654 (26, 25–27)	238 (4, 3–4)
Arawa	3183	439 (14, 13–15)	61 (2, 1–2)
Lemakot	9754	4111 (42, 41–43)	77 (0.7, 0.6–1.0)
Total	35,329	11,321 (32, 32–33)	674 (2, 1.8–2.1)

*RDT* rapid diagnostic test, *CI* confidence interval

**Fig. 2:**
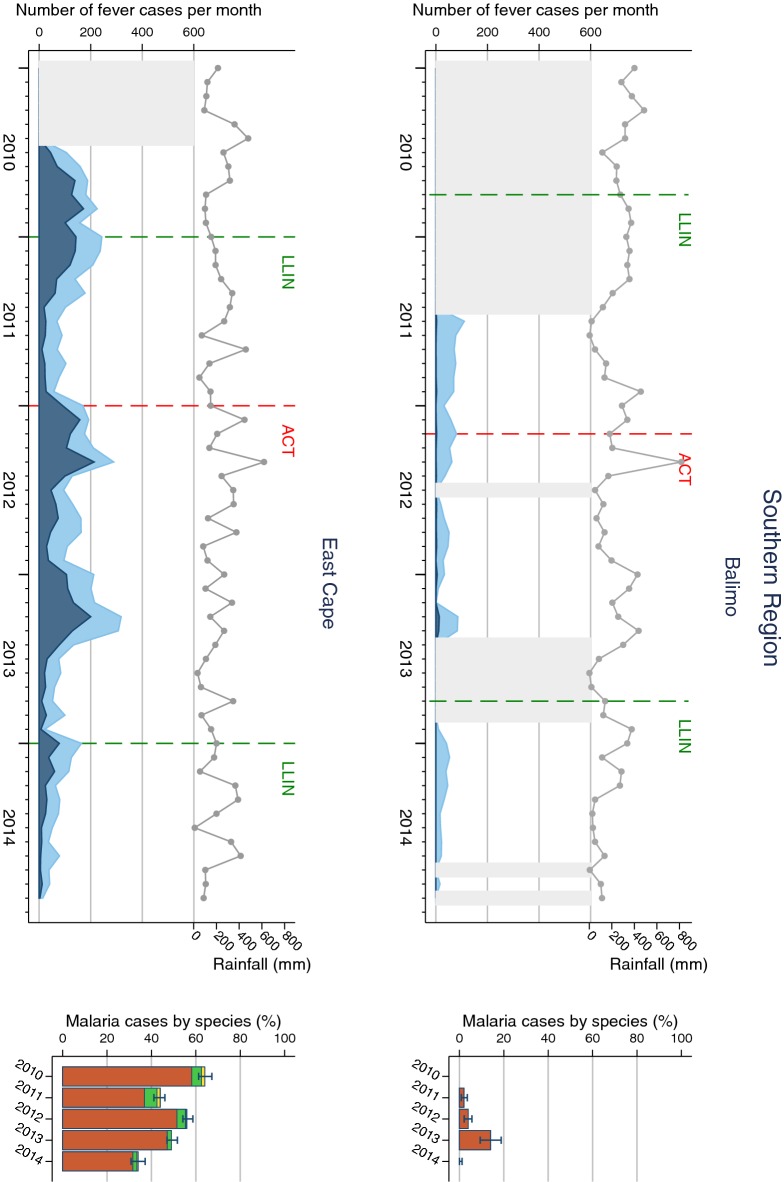
Malaria cases in Southern Region (Balimo and East Cape) sites. Left of each panel: monthly number of fever cases RDT negative (bright blue) and RDT positive (dark blue); cumulative monthly rainfall (grey line); timing of LLIN distribution and introduction of ACT (vertical dashed lines). Missing data is indicated by light grey shaded background. Right of each panel: annual RDT positivity (bar total) by species: *P. falciparum* (orange)*, P. vivax* (green), mixed infections (yellow), no species data available (white)

**Fig. 3:**
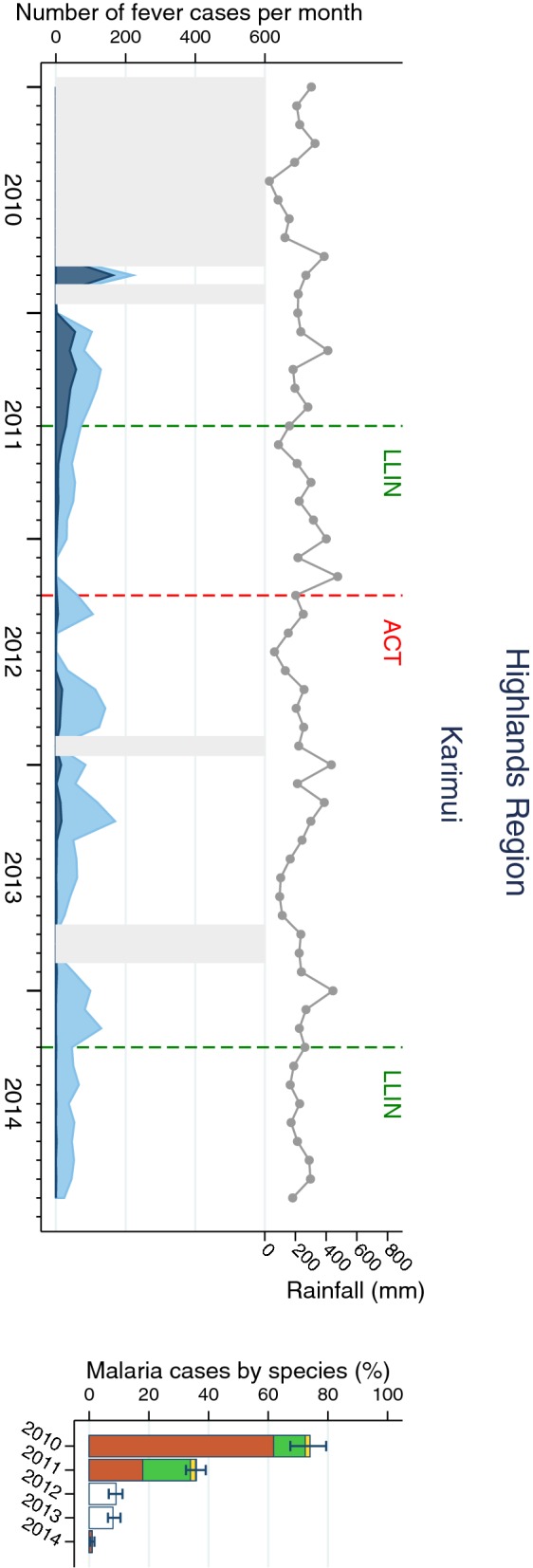
Malaria cases in Highlands Region (Karimui) site. Left of each panel: monthly number of fever cases RDT negative (bright blue) and RDT positive (dark blue); cumulative monthly rainfall (grey line); timing of LLIN distribution and introduction of ACT (vertical dashed lines). Missing data is indicated by light grey shaded background. Right of each panel: annual RDT positivity (bar total) by species: *P. falciparum* (orange*), P. vivax* (green), mixed infections (yellow), no species data available (white)

**Fig. 4:**
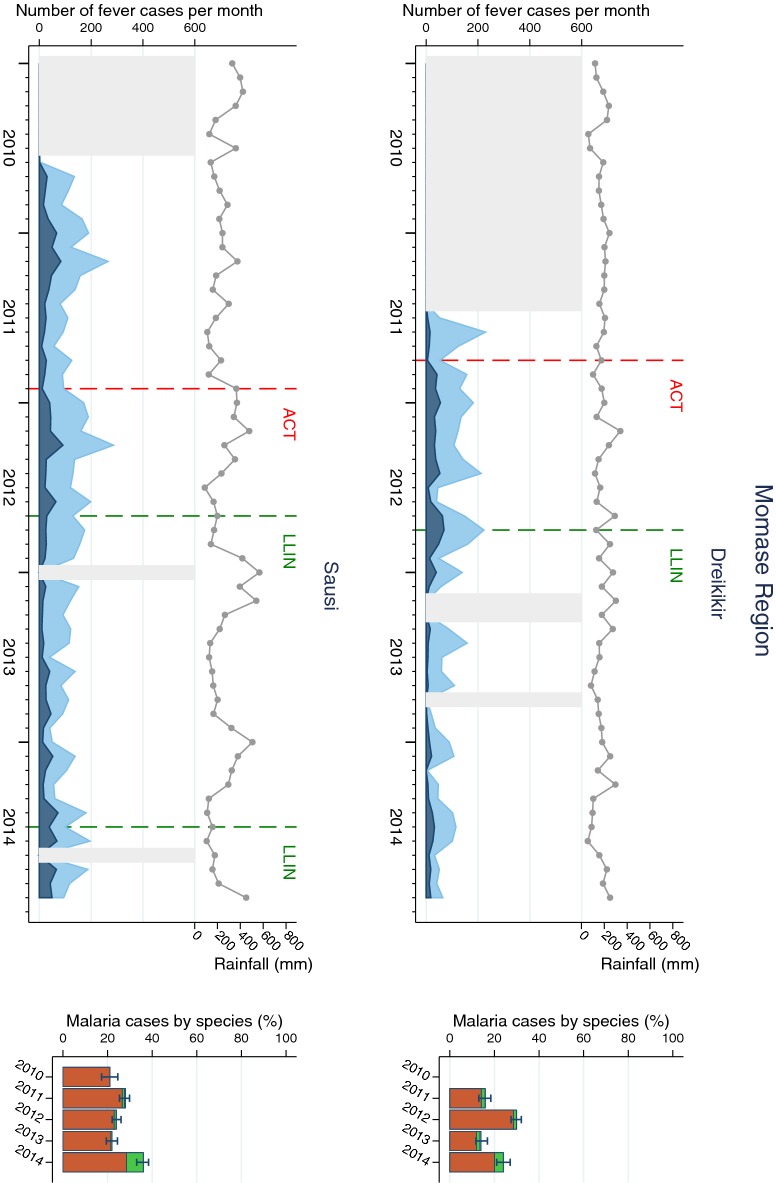
Malaria cases in Momase Region (Dreikikir and Sausi) sites. Left of each panel: monthly number of fever cases RDT negative (bright blue) and RDT positive (dark blue); cumulative monthly rainfall (grey line); timing of LLIN distribution and introduction of ACT (vertical dashed lines). Missing data is indicated by light grey shaded background. Right of each panel: annual RDT positivity (bar total) by species: *P. falciparum* (orange)*, P. vivax* (green), mixed infections (yellow), no species data available (white)

**Fig. 5:**
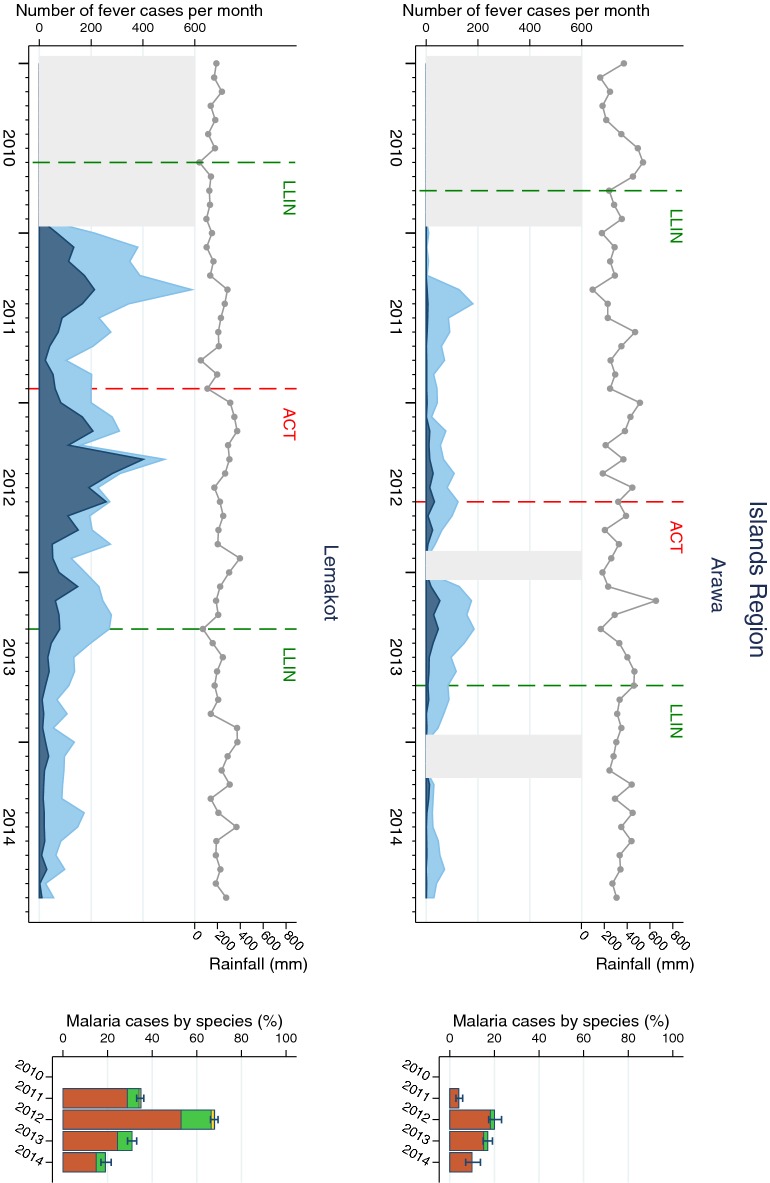
Malaria cases in Islands Region (Arawa and Lemakot) sites. Left of each panel: monthly number of fever cases RDT negative (bright blue) and RDT positive (dark blue); cumulative monthly rainfall (grey line); timing of LLIN distribution and introduction of ACT (vertical dashed lines). Missing data is indicated by light grey shaded background. Right of each panel: annual RDT positivity (bar total) by species: *P. falciparum* (orange)*, P. vivax* (green), mixed infections (yellow), no species data available (white)

The pattern of fever and malaria cases varied over the surveillance period and between SHFs (Figs. 2, 3, 4 and 5). All sites displayed monthly variations but there was no clear relationship with rainfall patterns. The number of fever and malaria cases decreased over the surveillance period in all sites except in Dreikikir and Sausi, where after an initial decrease an increase was noted in 2014. Malaria cases initially increased in Lemakot (2012), but decreased steadily thereafter. Annual RDT positivity decreased steadily over the surveillance period in most sites but fluctuations were observed especially in sites with low numbers of cases. A substantial increase in RDT positivity in Lemakot (from 35 to 68%) in the year 2012 with proportional increase in *P. vivax* and a spike of cases in women aged 15–20 (Additional file 6) suggests a local epidemic.

*Plasmodium falciparum* was the predominant species in all sites and all years even though species composition fluctuated over time and differed between sites. Balimo was the only SHF in which no infections with *P. vivax* were detected. Proportional increases of *P. vivax* were observed in Lemakot (2012) and Sausi (2014) at a time when the total number of malaria cases also increased (Figs. 2 and 5). Over the entire surveillance period, only 0.2% of malaria cases were diagnosed with *P. malariae* and 0.02% with *P. ovale*.

The annual incidence rate of malaria calculated for four sites ranged from 1/1000 population in Karimui in 2014 to 187/1000 in Lemakot in the peak year 2012. Incidence rates were highest in East Cape and Lemakot, except in 2014, when Sausi exhibited significantly higher incidence than the other sites. Severe malaria incidence ranged from 0.4/1000 in Karimui in 2014 to 28/1000 in Lemakot in 2011. In general, severe malaria incidence was highest in 2011 and lowest in 2014, except in Sausi, where a 2.6-fold increase was observed after 2013 (Table 2). The annual proportion of malaria cases being severe malaria ranged from 4% in East Cape in 2013 to 67% in Karimui in 2014. In Balimo and Arawa all severe malaria cases were attributed to *P. falciparum.* The greatest proportion of severe malaria with *P. vivax* (39%) was observed in Karimui in 2011 but by 2014 all cases were *P. falciparum* (Additional file 5). Since the number of severe malaria cases is very low in Karimui some of the variations might be attributed to chance fluctuations.

**Table 2:** Annual incidence of malaria and ‘severe malaria’ per 1000 population in four sentinel health facilities

Health facility	2010		2011		2012		2013		2014	
	N	95% CI	N	95% CI	N	95% CI	N	95% CI	N	95% CI
Malaria incidence										
East Cape	115	(107, 124)	117	(109, 125)	179	(170, 189)	141	(133,150)	47	(42, 52)
Karimui	19	(16, 22)	34	(31, 38)	6	(4, 8)	6	(5, 8)	1	(0.2, 1)
Sausi	20	(17, 24)	79	(72, 86)	79	(72, 87)	43	(38, 48)	79	(72, 86)
Lemakot	–	–	113	(107,119)	187	(180, 194)	55	(51, 59)	19	(17, 22)
‘Severe malaria’ incidence										
East Cape	7	(5, 9)	24	(20, 28)	10	(8, 13)	6	(4, 8)	3	(2, 5)
Karimui	2	(1, 4)	14	(12, 17)	3	(2, 4)	2	(1, 3)	0.4	(0.1, 1)
Sausi	1	(1, 3)	18	(15, 22)	13	(10, 16)	8	(6, 10)	21	(17, 25)
Lemakot	–	–	28	(25, 31)	27	(24, 30)	6	(5, 8)	4	(3, 5)

Only sites with available census denominator data were included

*CI* confidence interval

**Fig. 6:**
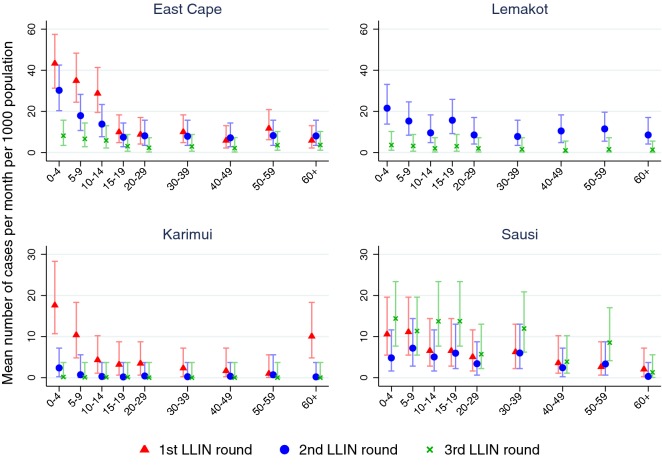
Malaria incidence rate by age group after each LLIN distribution round in four sites. *LLIN* long-lasting insecticide-treated bed net

The effect of the LLIN distribution on age-specific malaria incidence was assessed in the four sites with available age-specific population data. Malaria incidence was reduced with each LLIN distribution round in East Cape, Karimui and Lemakot. The greatest decrease was observed in the age groups 0–4 years and 5–9 years (Fig. 6). Incidence in Sausi initially decreased but increased again after the third distribution. When disaggregated by age group and gender, females in some sites and age groups appeared to have a higher incidence of malaria, e.g., in Lemakot (age group 15–19) and Sausi (age group 30–39), and differences in incidence rates between distribution rounds did not always affect males and females equally (Additional file 6).

Self-reported LLIN use increased in general with each distribution round and then gradually decreased over the subsequent years. Net use was highest in Sausi (90–100%), Balimo (95–100%) and Dreikikir (77–86%) and lowest in the two Islands sites of Arawa (21–69%) and Lemakot (41–48%), confirming data of the 2010/2011 national malaria indicators survey (Additional file 7) [18]. The treatment of malaria patients with ACT was consistently high (> 80% each year) after the introduction of the drug in Balimo, East Cape, Dreikikir, Sausi, and Lemakot. The previous treatment combination of amodiaquine or chloroquine plus sulfadoxine–pyrimethamine (SP) was phased out over the same period. The opposite trend was observed in Karimui, where ACT was gradually substituted by the previous treatment regimen 1 year after its introduction and in Arawa, where in 2014, most patients were treated neither with the old, nor with the new regimen (Additional file 8). Annually, less than 1.3% of negative cases were treated with ACT in all SHFs. Information on the use of primaquine is available in the Additional file 9.

Due to significant heterogeneity in the estimated effects of LLIN distribution rounds on the number of malaria cases between sites (p < 0.001, interaction test), the effects of interventions were estimated for each SHF individually and adjusted for El Niño/La Niña years. In general, subsequent distribution of LLINs led to cumulative reductions in the number of malaria cases (Table 3). The greatest reductions were in settings where the number of cases was the lowest (Balimo, Karimui, Arawa) rather than in places with high net use but high case load. In Sausi, where an earlier study had suggested significant impact of the first LLIN distribution [9], the second distribution reduced the number of cases by 57% (95% CI 11–79%) but a two-fold increase was observed after the third distribution.

**Table 3:** Estimated effects of each round of LLIN distribution and ACT introduction on the number of malaria cases by sentinel health facility

Health facility	2nd vs. 1st LLIN distribution		3rd vs. 2nd LLIN distribution		Introduction of ACT	
	IRR	95% CI	IRR	95% CI	IRR	95% CI
Balimo	–	–	1.32E−09	(0.00, –)	1.21	(0.47, 3.14)
East Cape	0.53	(0.20, 1.41)	0.34	(0.18, 0.64)	1.50	(0.79, 2.85)
Karimui	0.19	(0.06, 0.58)	0.12	(0.03, 0.43)	0.95	(0.32, 2.80)
Dreikikir	1.15	(0.54, 2.46)	–	–	3.95	(1.89, 8.25)
Sausi	0.43	(0.21, 0.89)	2.08	(1.16, 3.72)	1.28	(0.76, 2.16)
Arawa	–	–	0.15	(0.06, 0.36)	1.79	(0.57, 5.64)
Lemakot	–	–	0.26	(0.14, 0.50)	1.73	(1.09, 2.75)

All estimates were adjusted for La Niña or El Niño year

*IRR* incidence rate ratio, *CI* confidence interval

The change of treatment from amodiaquine or chloroquine plus SP to ACT did not appear to significantly affect the number of malaria cases except in Drekikir and Lemakot where an increase was observed.

## Discussion

Malaria surveillance in SHFs revealed varying trends in the number of malaria cases and the magnitude of their association with control interventions between 2010 and 2014. In general, reductions in the number of malaria cases were observed with each of three rounds of household-level LLIN distribution while no substantial reductions followed the change of treatment to ACT. The number of malaria cases was found to increase in one site after the third distribution round. The findings disclose a substantial sub-national heterogeneity in the epidemiology and control of malaria in PNG.

After the first large-scale LLIN distribution in PNG, data from six sentinel surveillance sites indicated a drop in the average monthly malaria incidence rate from 13/1000 population to 2/1000 (incidence rate ratio = 0.12; 95% CI 0.09–0.17) and reductions in prevalence and transmission confirming significant short-term effect of LLIN in the absence of ACT [9]. Previous modelling studies suggested that the effect of LLIN may fade over time as acquired immunity in the population is reduced, particularly in areas with a high pre-intervention entomological inoculation rate [19]. The situation observed in Dreikikir and Sausi is consistent with this prediction. On the other hand, considering LLIN coverage was consistently high (self-reported use 90–100% in Sausi, 77–89% in Dreikikir, Additional file 5) [9], other factors such as aging of nets may fuel ongoing transmission. In the absence of insecticide resistance, early and outdoor biting of *Anopheles* mosquitoes has been identified as a threat to the effectiveness of LLINs. Entomological studies in Sausi have described a shift in mosquito biting to earlier hours following the first LLIN distribution (the peak exposure time to infectious bites shifted from later than 9 p.m. in 2008 to between 6 and 7 p.m. in 2011) resulting in decreased protection against mosquito bites [10, 20].

Gender differences, or anomalies in the age-specific incidence rates, (e.g., higher incidence rates in females age 15–19 in Lemakot; Additional file 6) might suggest gender-specific risk. In Lemakot, the substantial increase in malaria cases in 2012 was disproportionately due to cases in teenage girls, suggesting a local outbreak. Age or gender-specific behaviour (e.g., evening activities, division of household chores) or other social or cultural determinants including location and quality of houses may result in different levels of exposure. Such factors have been well investigated in settings in which most of the transmission is limited to specific population groups (e.g., in Southeast Asia [21–23]). In highly diverse settings such as PNG, one challenge will be to identify risk factors that disproportionately affect particular population groups, and to translate such knowledge into targeted control action. A mixed methods approach that captures behavioural patterns alongside prevalence and incidence data, linked with entomological investigations in settings with ongoing transmission, will be an important first step.

Most of the evidence of the impact of insecticide-treated nets originates from African settings and is limited to the effect on *P. falciparum* [24]. While multiple studies demonstrated the effect of LLIN programmes on *P. falciparum* incidence (e.g. [25, 26]), evidence from settings with several *Plasmodium* species is scarce. Although it has been suggested that the impact of vector control on *P. vivax* may be delayed due to the parasite’s biology, this study only found short-term transient increases in the proportion of *P. vivax* by light microscopy, suggesting that at intermediate to high transmission, both species are affected by vector control and both species may resurge. A modelling study found that in such areas LLINs alone could not lead to interruption of *P. vivax* transmission and additional tools are required to accelerate to elimination [27]. Considering the abundance of low level parasitaemia, particularly in *P. vivax* infections as transmission is reduced, more sensitive diagnostic tools may need to be applied to monitor progress and species composition [28].

A number of field and modelling studies have shown a reduction in malaria cases and/or transmission after the introduction of ACT alone and in combination with LLINs [29–34]. In this study, the change in first-line treatment from amodiaquine or chloroquine plus SP to ACT did not lead to a decrease in the number of cases. Neither did it result in an increase in the proportion of *P. vivax* cases despite the higher susceptibility of *P. falciparum* to artemether-lumefantrine [35] and the low consistent use of primaquine as radical cure of *P. vivax*. In general, the previous treatment was used widely (though not always strictly according to guidelines) before the introduction of ACT (Additional file 7) and the regimen had retained approximately 82% efficacy (chloroquine plus SP in 2005–2007 [35]) limiting the increase in efficacy after ACT roll-out to 13%. Additionally, while an efficacious drug can improve clinical outcomes [36] a community-wide effect on transmission (and hence incidence) is, among other factors, a function of treatment seeking and prevalence of asymptomatic and sub-microscopic infections. Individuals with asymptomatic infections fuel ongoing transmission and do not seek treatment [8, 37, 38]. In PNG, only 43% of fever cases were found to have attended a health facility (2013/14) [39], thus the increase in efficacy might have been insufficient to translate into reduced transmission and incidence, as already demonstrated in an African high-transmission setting [40]. In the two sites in which an increase in the number of malaria cases was observed following the introduction of ACT, overall facility attendance did not suggest an availability effect resulting from the introduction of free intervention, as documented elsewhere [41, 42]. A recent study in PNG even suggests that the shift to test-based ACT treatment may have negatively impacted treatment seeking and patient satisfaction, possibly related to low perceived quality of care provided to patients with non-malarial illness [43]. Differences in the length of post-intervention periods used for the regression model may have affected the reliability of estimates for Dreikikir as the period preceding ACT introduction was only 3 months (Additional file 2), most likely not enough to gain reliable estimates for both interventions.

Different surveillance starting points and lack of pre-LLIN data were limitations to this study but data from previous surveillance activities and national prevalence surveys provide supporting evidence of the short-term effect of LLINs [4, 8, 9]. Fluctuations in treatment seeking or facility attendance may have influenced incidence estimates to a certain degree, but data from repeat national surveys suggests that the proportion of fever patients attending a formal health facility has remained largely unchanged since the first assessment in 2008/09 [44].

Despite previous validation and use in other settings [14, 45], none of the available site-specific satellite weather variable (EVI: MODIS products MOD13Q1 and MOD13A3; and rainfall: TRMM product 3B43) could explain variations in malaria incidence over time which supports historical descriptions of an intricate and complex environment driving malaria epidemiology across in PNG [2, 5, 8, 46, 47]. Particularly in an environment with high overall rainfall, weather variables may not be a good single predictor of malaria incidence. In contrast the El Niño/La Niña phenomena appeared to be a useful and more stable environmental predictor since it affects larger areas for a longer period of time than site-specific weather data.

Differences in pre-intervention malaria transmission and in the impact of interventions between sites are a function of the diverse social and ecological settings which lead to differences in vector abundance, vector behaviour and human–vector interaction. Multi-disciplinary studies on a smaller scale are required to broaden the understanding of malaria transmission dynamics on a sub-national level and identify regional factors driving the observed heterogeneity. Insights from such investigations should be translated into response strategies that take into consideration sub-national heterogeneity in the drivers of ongoing malaria transmission. A robust surveillance system reporting case incidence should be supported by monitoring of entomological and immunological parameters to explain differences in the impact of interventions.

## Conclusions

Subsequent household level distributions of LLINs had a cumulative effect on reducing the number of malaria cases in the SHFs but the magnitude of the association varied between sites and over time. Changing treatment to ACT had no apparent effect. Malaria programmes covering diverse transmission settings such as PNG must consider local heterogeneity when choosing interventions and ensure continuous monitoring of trends.

## Supplementary information

**Additional file 1.** Sentinel sites description.

**Additional file 2.** Timeline of implementation of malaria control interventions and surveillance in each sentinel health facility, 2005–2014.

**Additional file 3.** Rapid diagnostic test and light microscopy results.

**Additional file 4.** Key environmental variables by site.

**Additional file 5.** Severe malaria in sentinel health facilities.

**Additional file 6.** Malaria incidence by age group and sex.

**Additional file 7.** Self-reported net use.

**Additional file 8.** Percentage of malaria cases treated with the previous first line treatment or partial treatment (mono-therapy), and with artemisinin-based combination therapy.

**Additional file 9.** Percentage of malaria cases treated with primaquine (for different RDT results), and with artemisinin-based combination therapy.

## Data Availability

The datasets used and/or analysed during the current study are available from the corresponding author on reasonable request.

## Abbreviations

ACT: artemisinin-based combination therapy
CI: confidence interval
DDT: dichlorodiphenyltrichloroethane
EOS: Earth Observing System
EVI: Enhanced Vegetation Index
GES DISC: Goddard Earth Sciences Data and Information Services Center
Hb: haemoglobin
IRR: incidence rate ratio
LLIN: long-lasting insecticidal net
NASA: National Aeronautics and Space Administration
NMCP: National malaria control programme
PNG: Papua New Guinea
PNGIMR: Papua New Guinea Institute of Medical Research
RDT: rapid diagnostic test
SHF: sentinel health facilities
SP: sulfadoxine–pyrimethamine
TRMM: Tropical Rainfall Measuring Mission
