# Supplementary material for: Repeated mosquito net distributions, improved treatment, and trends in malaria cases in sentinel health facilities in Papua New Guinea
Source: Malar J. 2019 Nov 12;18:364. doi: 10.1186/s12936-019-2993-6 (PMC6852945; doi:10.1186/s12936-019-2993-6)
Supplement: Supplementary file 1 — Additional file 1. Sentinel sites description. [file 12936_2019_2993_MOESM1_ESM.docx]

**Additional file 1: Sentinel sites description^[[1]](#footnote-1)^**

| **Health facility** | **Region** | **Province** | **Altitude (m)** | **Population (villages)** | **Health facility surveillance** | **Census** |
| --- | --- | --- | --- | --- | --- | --- |
| **Balimo** | Southern | Western | 15 | NA | 06/2011-12/2014 | NA |
| **East Cape** | Southern | Milne Bay | 15 | 5,684*  (509) | 02/2010-12/2014 | 2010 |
| **Karimui** | Highlands | Chimbu (Simbu) | 1140 | 8,506*  (24) | 11/2010-12/2014 | 2010 |
| **Dreikikir** | Momase | East Sepik | 420 | 24,359**  (NA) | 06/2011-12/2014 | NA |
| **Sausi** | Momase | Madang | 170 | 5,158*  (48) | 08/2010-12/2014 | 2010 |
| **Arawa** | Islands | Bougainville | 10 | 15,447**  (NA) | 01/2011-12/ 2014 | NA |
| **Lemakot** | Islands | New Ireland | 15 | 10,384*  (33) | 01/2011-12/2014 | 2010 |

NA Not available data *Study census **Provided by District Health Authorities 2014

**Details of each site**

East Cape is located in Milne Bay Province which comprises only 14% of land mass with the rest being ocean; 25% of its landmass are islands and atolls ^[[2]](#footnote-2)^. Karimui is in a highland fringe area in Simbu Province. About 70% of Simbu Province lies at altitudes between 1000-2600 m above sea level and normally has a very wet and non-seasonal climate ^[[3]](#footnote-3)^,^[[4]](#footnote-4)^ Lemakot is located on the main island of New Ireland Province, which is long (~200 km), narrow (~8 km) and mountainous with a wet tropical climate ^[[5]](#footnote-5)^. Sausi is located in Madang Province in the Ramu Valley where industrial sugar cane and oil palm estates have been established. Nearby areas are regularly flooded by the Ramu River and its branches. Arawa is located in the centre of the Autonomous Region of Bougainville. The climate in Bougainville is tropical with consistent temperature, rainfall and humidity throughout the year ^[[6]](#footnote-6)^. Balimo is located in Western Province along the Aramia River and the surrounding lowland forest. Lagoon systems with seasonal flooding surround villages situated on land and islands points ^[[7]](#footnote-7)^,^[[8]](#footnote-8)^. Dreikikir is located in East Sepik Province in hilly terrain (~200 m). The climate in East Sepik is seasonal with most abundant rainfall between December and June^[[9]](#footnote-9),^^[[10]](#footnote-10),^^[[11]](#footnote-11),^^[[12]](#footnote-12)^.

1. Manuel W Hetzel, Justin Pulford, and others, ‘Evaluation of the Global Fund-Supported National Malaria Control Program in Papua New Guinea, 2009-2014’, *PNG Med J*, 57.1–4 (2014), 7–29. [↑](#footnote-ref-1)
2. R M Bourke, M G Allen, and J G Salisbury, ‘Food Security for Papua New Guinea’, in *Proceedings of the Papua New Guinea Food and Nutrition 2000 Conference, PNG University of Technology, Lae* (Lae, Papua New Guinea, 2000), pp. 1–882. [↑](#footnote-ref-2)
3. Robert D. Attenborough and Michael P. Alpers, *Human Biology in Papua New Guinea : The Small Cosmos* (Oxford University Press, 1992). [↑](#footnote-ref-3)
4. Bourke, Allen, and Salisbury. [↑](#footnote-ref-4)
5. David K. Holdsworth, Chris L. Hurley, and Sue E. Rayner, ‘Traditional Medicinal Plants of New Ireland, Papua New Guinea’, *Quarterly Journal of Crude Drug Research*, 18.3 (1980), 131–39. [↑](#footnote-ref-5)
6. CM Yule, ‘Trophic Relationships and Food Webs of the Benthic Invertebrate Fauna of Two Aseasonal Tropical Streams on Bougainville Island, Papua New Guinea’, *Journal of Tropical Ecology*, 12.4 (1996), 517–34. [↑](#footnote-ref-6)
7. Jeffrey Mitchell Warner, ‘The Epidemiology of Melioidosis in Papua New Guinea. PhD Thesis, James Cook University’, 2004. [↑](#footnote-ref-7)
8. Jeffrey Mitchell Warner and others, ‘Melioidosis in a Rural Community of Western Province, Papua New Guinea’, *Trans R Soc Trop Med Hyg*, 101 (2007), 809–13. [↑](#footnote-ref-8)
9. Moses J Bockarie and Henry Dagoro, ‘Are Insecticide-Treated Bednets More Protective against Plasmodium Falciparum than Plasmodium Vivax-Infected Mosquitoes?’, *Malaria Journal*, 5 (2006), 15. [↑](#footnote-ref-9)
10. Manuel W Hetzel, Susan Paul, and others, ‘Proportion of Fevers Attributable to Malaria Varies Significantly between Sites in Papua New Guinea’, *PNG Med J*, 57.1–4 (2014), 39–51. [↑](#footnote-ref-10)
11. Moses J Bockarie and others, ‘Randomised Community-Based Trial of Annual Single-Dose Diethylcarbamazine with or without Ivermectin against Wuchereria Bancrofti Infection in Human Beings and Mosquitos’, *Lancet*, 351.9097 (1998), 162–68. [↑](#footnote-ref-11)
12. James W Kazura and others, ‘Parasitologic and Clinical Features of Bancroftian Filariasis in a Community in East Sepik Province, Papua New Guinea’, *American Journal of Tropical Medicine and Hygiene*, 33.6 (1984), 1119–23. [↑](#footnote-ref-12)
