# Supplementary material for: Repeated mosquito net distributions, improved treatment, and trends in malaria cases in sentinel health facilities in Papua New Guinea
Source: Malar J. 2019 Nov 12;18:364. doi: 10.1186/s12936-019-2993-6 (PMC6852945; doi:10.1186/s12936-019-2993-6)

**Additional file 2: Timeline of implementation of malaria control interventions and surveillance in each sentinel health facility, 2005-2014**


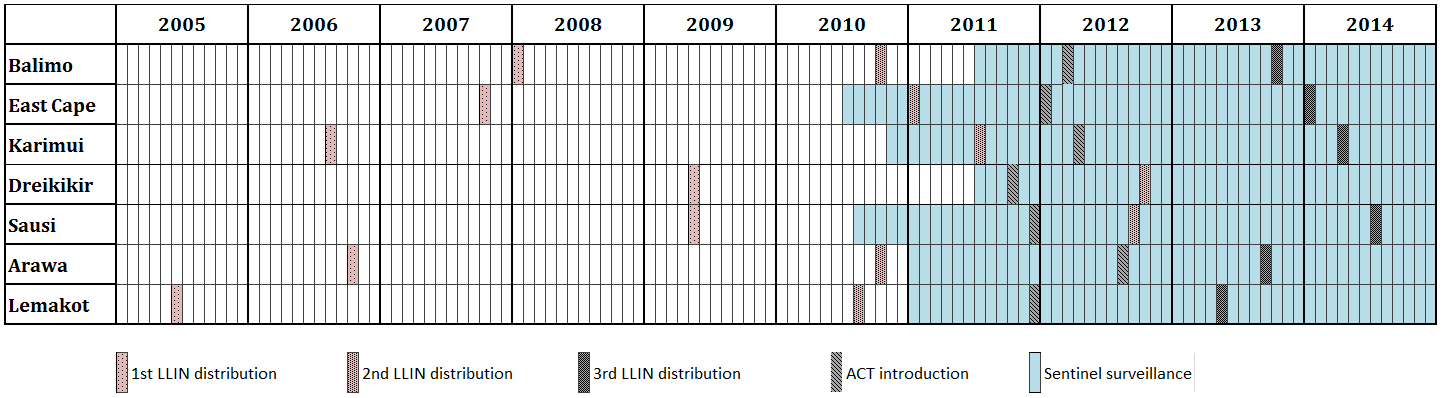

Supplement: Supplementary file 2 — Additional file 2. Timeline of implementation of malaria control interventions and surveillance in each sentinel health facility, 2005–2014. [file 12936_2019_2993_MOESM2_ESM.docx]
