# Supplementary material for: Repeated mosquito net distributions, improved treatment, and trends in malaria cases in sentinel health facilities in Papua New Guinea
Source: Malar J. 2019 Nov 12;18:364. doi: 10.1186/s12936-019-2993-6 (PMC6852945; doi:10.1186/s12936-019-2993-6)
Supplement: Supplementary file 3 — Additional file 3. Rapid diagnostic test and light microscopy results. [file 12936_2019_2993_MOESM3_ESM.docx]

**Additional file 3: Rapid diagnostic test and light microscopy results**

Number of RDT-positive and RDT-negative cases and proportion of RDT results read by microscopy (for species identification)

|  | **2010** | | **2011** | | **2012** | | **2013** | | **2014** | |
| --- | --- | --- | --- | --- | --- | --- | --- | --- | --- | --- |
| **Health facility** | N positive | N negative | N positive | N negative | N positive | N negative | N positive | N negative | N positive | N negative |
|  | RDT (LM%) | RDT(LM%) | RDT(LM%) | RDT(LM%) | RDT(LM%) | RDT(LM%) | RDT(LM%) | RDT(LM%) | RDT(LM%) | RDT(LM%) |
| **East Cape** | 655(26) | 364(54) | 685(51) | 888(83) | 1083(29) | 837(70) | 879(15) | 902(45) | 299(19) | 582(62) |
| **Karimui** | 163(86) | 58(84) | 301(35) | 542(89) | 54(0) | 567(56) | 59(0) | 657(50) | 6(17) | 725(38) |
| **Lemakot** | NA | NA | 1205(65) | 2276(96) | 2062(21) | 975(66) | 623(11) | 1387(53) | 221(29) | 928(48) |
| **Sausi** | 104(25) | 397(37) | 418(51) | 1101(89) | 434(30) | 1375(59) | 241(17) | 861(49) | 457(29) | 824(54) |
| **Arawa** | NA | NA | 29(24) | 682(99) | 156(7) | 617(47) | 219(30) | 1071(55) | 35(9) | 313(73) |
| **Balimo** | NA | NA | 9(33) | 465(99) | 18(17) | 479(80) | 30(3) | 191(42) | 0(0) | 304(66) |
| **Dreikikir** | NA | NA | 116(64) | 629(98) | 465(44) | 1099(71) | 104(26) | 631(45) | 191(26) | 607(39) |

RDT = Rapid diagnostic test

LM% = Proportion of RDT-result read by light microscopy
