# Supplementary figures and images for: Repeated mosquito net distributions, improved treatment, and trends in malaria cases in sentinel health facilities in Papua New Guinea
Source: Malar J. 2019 Nov 12;18:364. doi: 10.1186/s12936-019-2993-6 (PMC6852945; doi:10.1186/s12936-019-2993-6)

**Additional file 4: Key environmental variables by site**


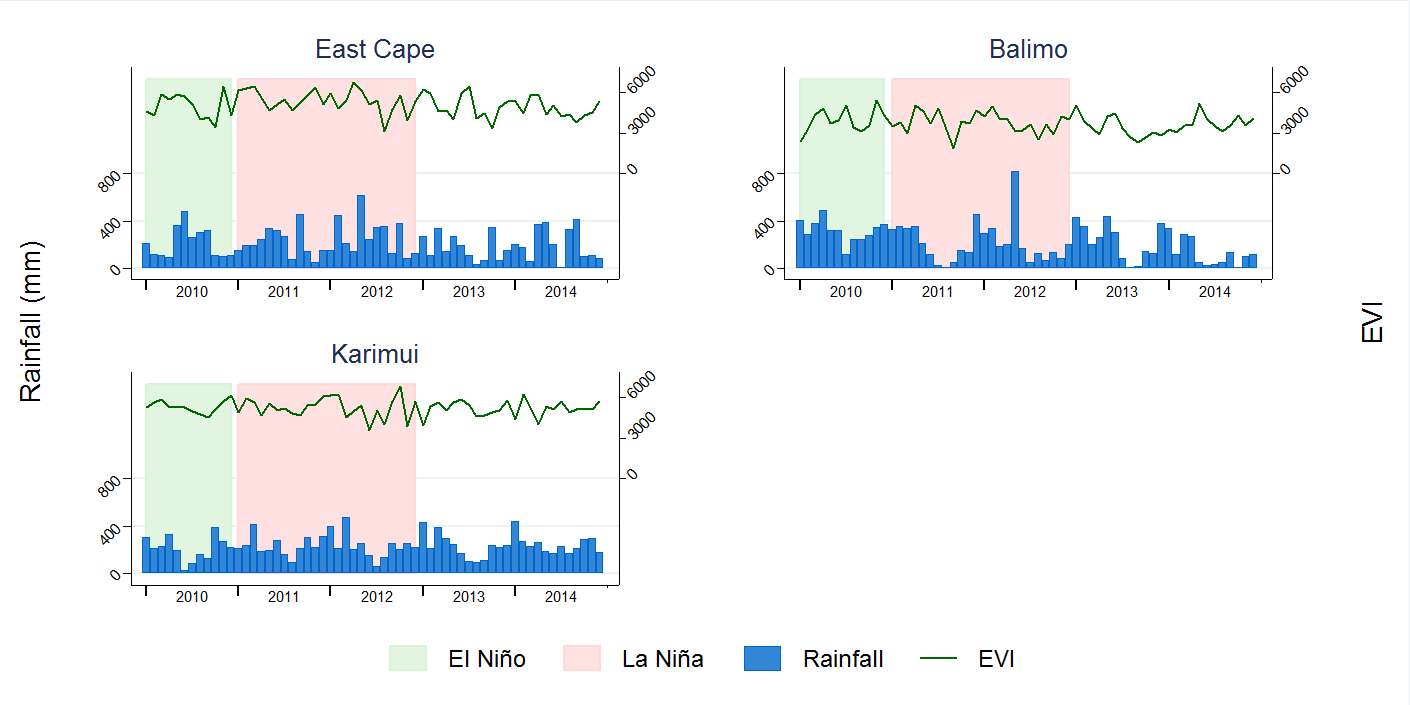


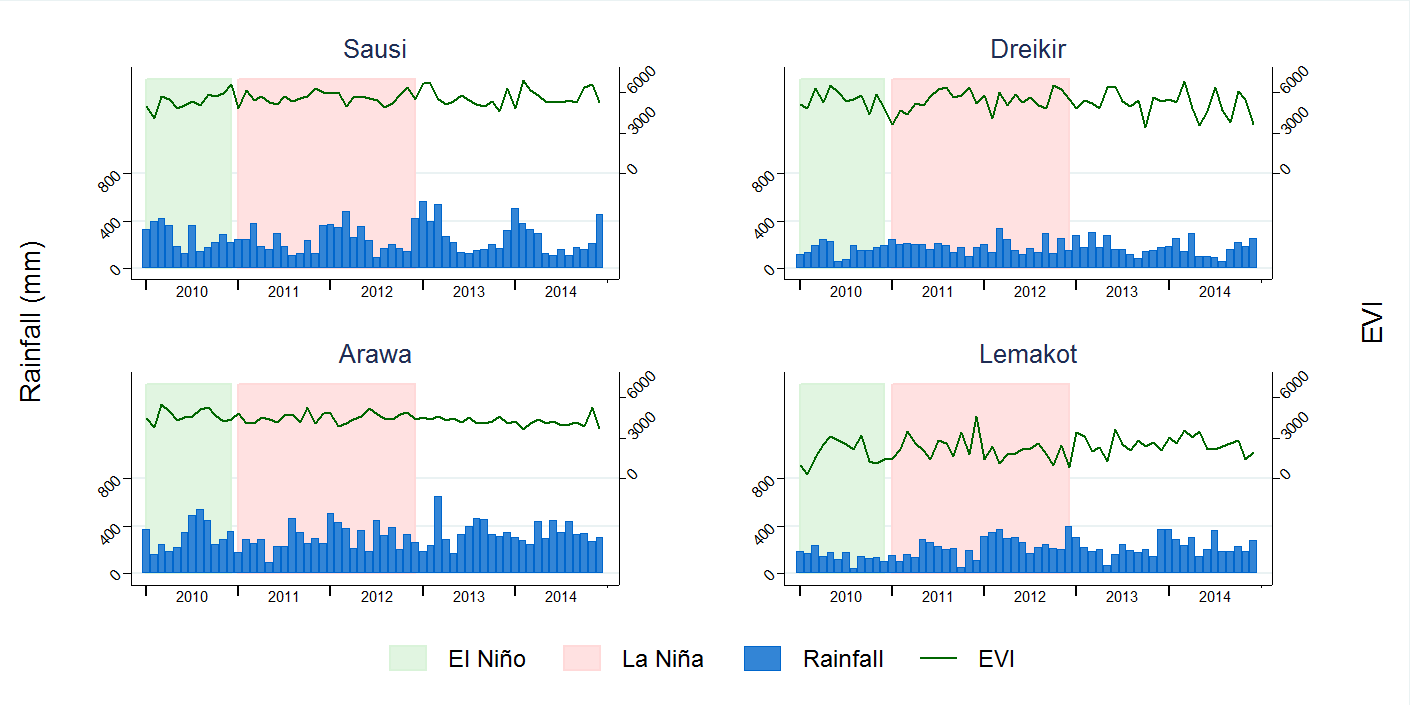

Supplement: Supplementary file 4 — Additional file 4. Key environmental variables by site. [file 12936_2019_2993_MOESM4_ESM.docx]
