# Supplementary material for: Repeated mosquito net distributions, improved treatment, and trends in malaria cases in sentinel health facilities in Papua New Guinea
Source: Malar J. 2019 Nov 12;18:364. doi: 10.1186/s12936-019-2993-6 (PMC6852945; doi:10.1186/s12936-019-2993-6)
Supplement: Supplementary file 5 — Additional file 5. Severe malaria in sentinel health facilities. [file 12936_2019_2993_MOESM5_ESM.docx]

**Additional file 5: Severe malaria in sentinel health facilities**


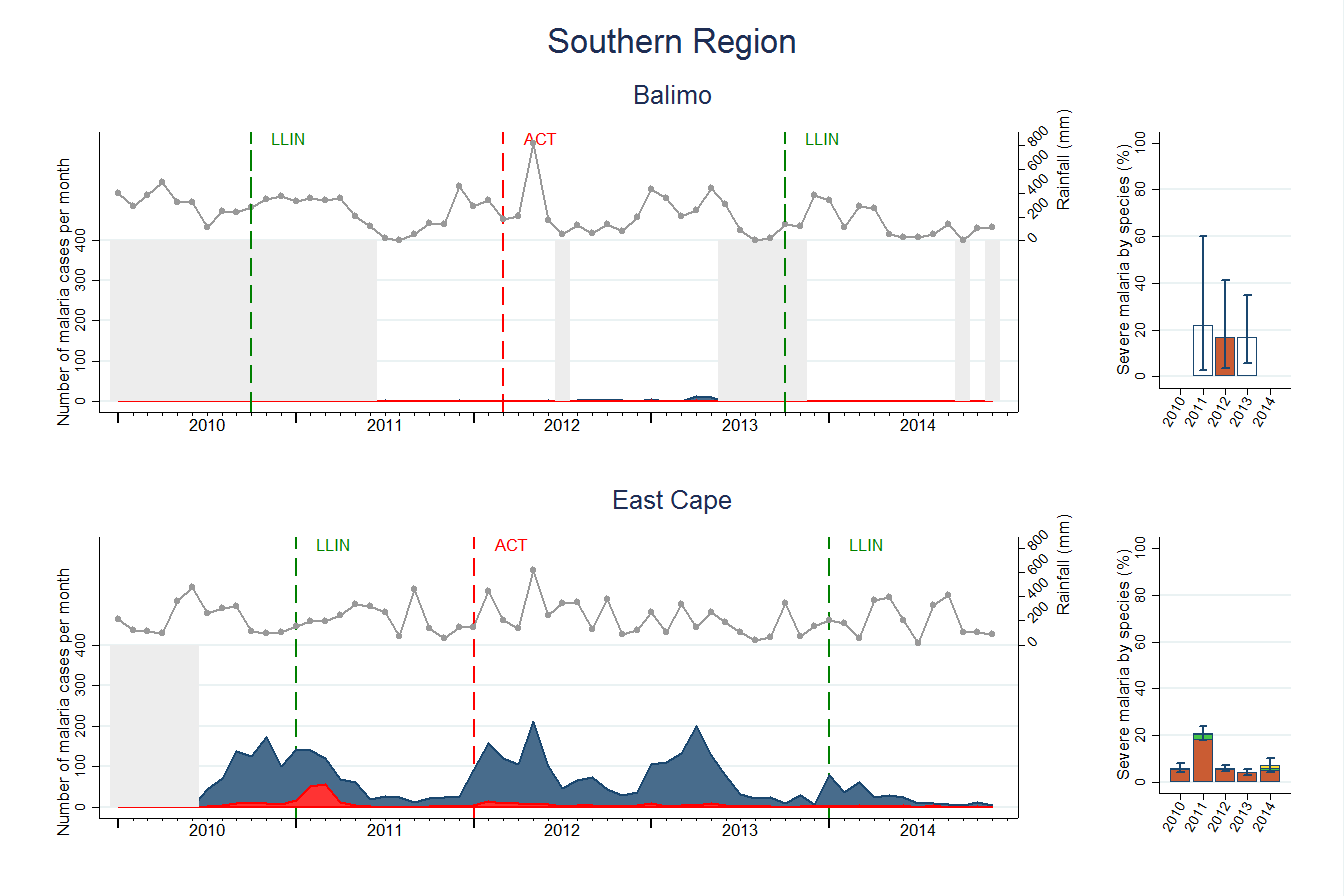


Severe malaria cases in Southern Region (Balimo and East Cape) sites. Left of each panel: monthly number of malaria cases; uncomplicated malaria (dark blue) and severe malaria (red); accumulated monthly rainfall (grey line); timing of LLIN roll-out and introduction of ACT (vertical dashed lines). Right of each panel: Percentage of malaria cases identified as severe (bar total) and Plasmodium species composition: P. falciparum (orange), P. vivax (green), mixed infections (yellow), no species data available (white)


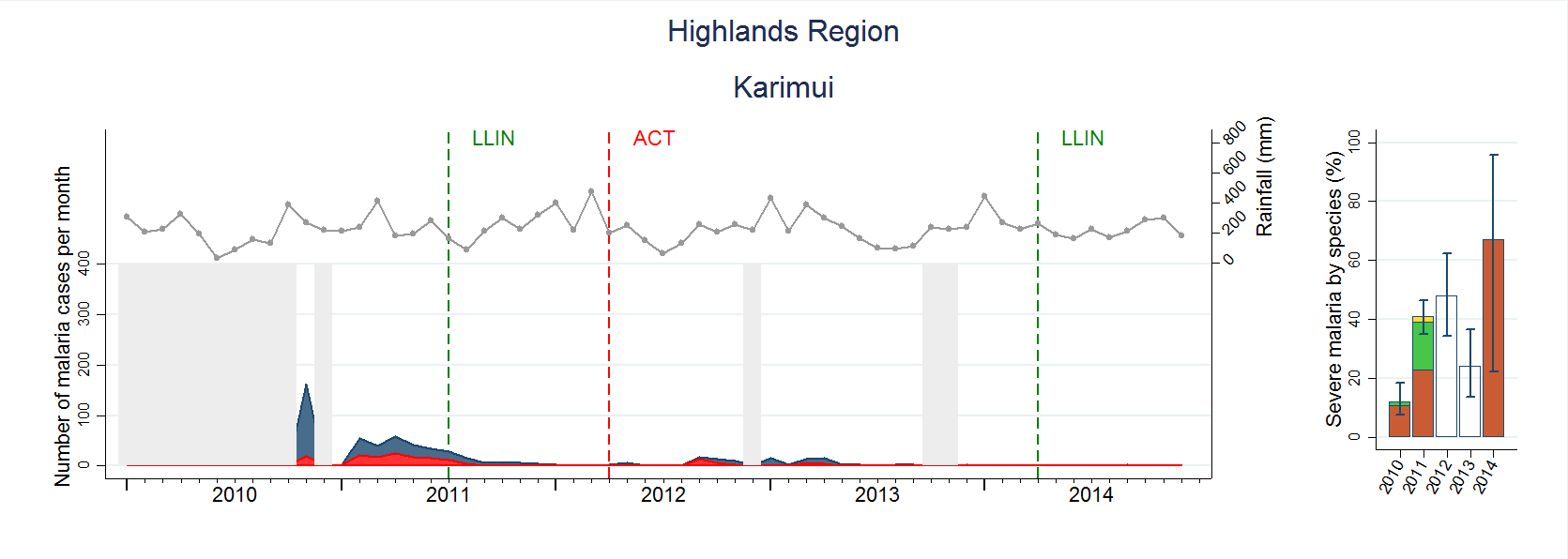


Severe malaria cases in Highlands Region (Karimui). Left of each panel: monthly number of malaria cases; uncomplicated malaria (dark blue) and severe malaria (red); accumulated monthly rainfall (grey line); timing of LLIN roll-out and introduction of ACT (vertical dashed lines). Right of each panel: Percentage of malaria cases identified as severe (bar total) and Plasmodium species composition: P. falciparum (orange), P. vivax (green), mixed infections (yellow), no species data available (white)


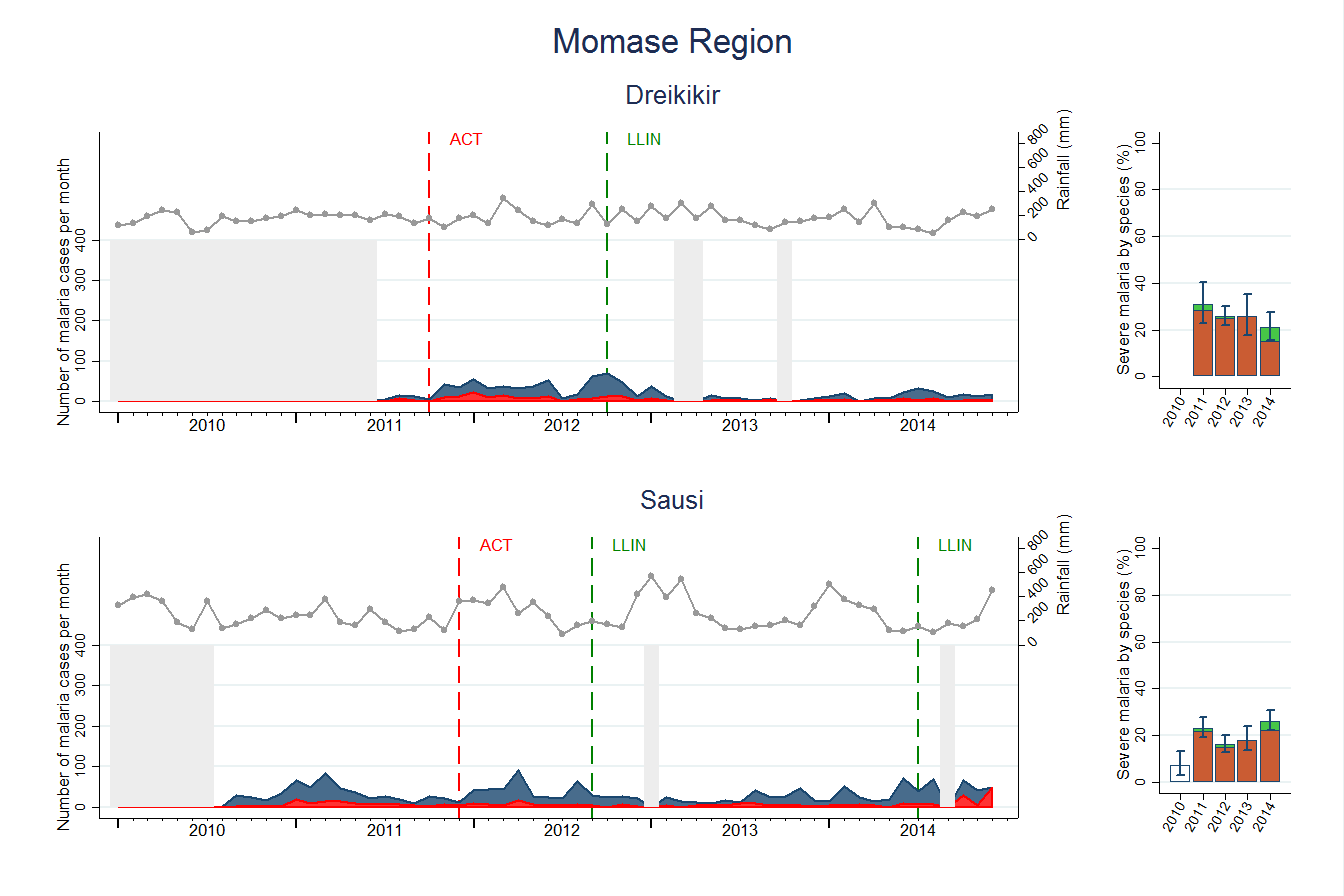


Severe malaria cases in Momase Region (Dreikikir and Sausi) sites. Left of each panel: monthly number of malaria cases; uncomplicated malaria (dark blue) and severe malaria (red); accumulated monthly rainfall (grey line); timing of LLIN roll-out and introduction of ACT (vertical dashed lines). Right of each panel: Percentage of malaria cases identified as severe (bar total) and Plasmodium species composition: P. falciparum (orange), P. vivax (green), mixed infections (yellow), no species data available (white)


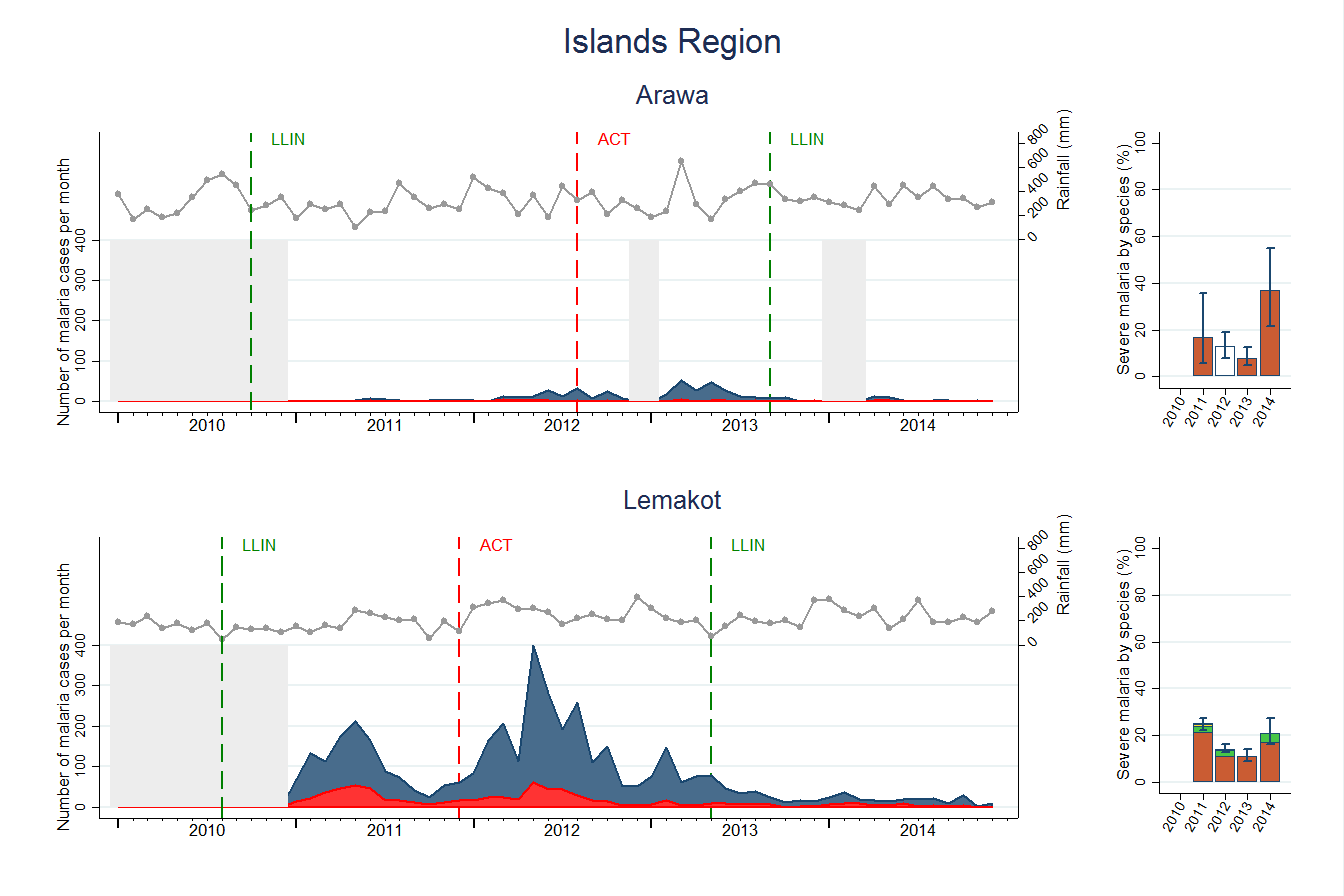


Severe malaria cases in Islands Region (Arawa and Lemakot) sites. Left of each panel: monthly number of malaria cases; uncomplicated malaria (dark blue) and severe malaria (red); accumulated monthly rainfall (grey line); timing of LLIN roll-out and introduction of ACT (vertical dashed lines). Right of each panel: Percentage of malaria cases identified as severe (bar total) and Plasmodium species composition: P. falciparum (orange), P. vivax (green), mixed infections (yellow), no species data available (white)
