# Supplementary material for: Repeated mosquito net distributions, improved treatment, and trends in malaria cases in sentinel health facilities in Papua New Guinea
Source: Malar J. 2019 Nov 12;18:364. doi: 10.1186/s12936-019-2993-6 (PMC6852945; doi:10.1186/s12936-019-2993-6)
Supplement: Supplementary file 6 — Additional file 6. Malaria incidence by age group and sex. [file 12936_2019_2993_MOESM6_ESM.docx]

**Additional file 6: Malaria incidence by age group and sex**

**
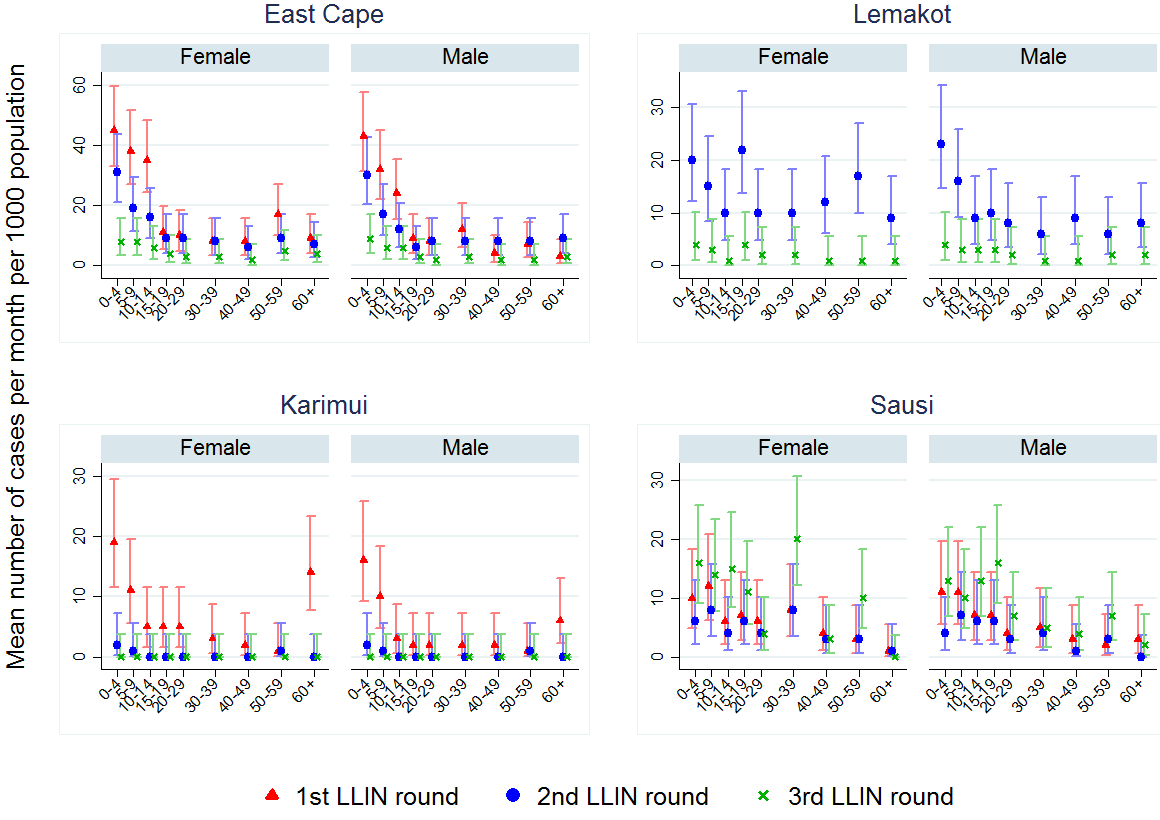
**Malaria incidence by age group and sex for each LLIN distribution round in four sites.

LLIN = long-lasting insecticidal nets
