# Supplementary material for: Repeated mosquito net distributions, improved treatment, and trends in malaria cases in sentinel health facilities in Papua New Guinea
Source: Malar J. 2019 Nov 12;18:364. doi: 10.1186/s12936-019-2993-6 (PMC6852945; doi:10.1186/s12936-019-2993-6)
Supplement: Supplementary file 7 — Additional file 7. Self-reported net use. [file 12936_2019_2993_MOESM7_ESM.docx]

**Additional file 7: Self-reported net use**

**
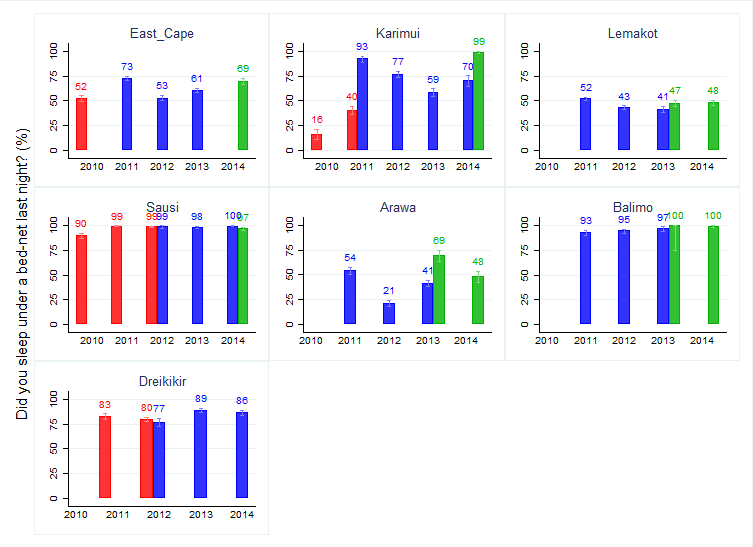
**

Self-reported net use by year and LLIN distribution round (red: after 1st round, blue: after 2nd round, green: after 3rd round).

For comparison, 2010/11 national malaria indicator survey net use results:

Southern Region 67% (95% CI: 57, 76)

Highlands Region 40% (29, 51)

Momase Region 49% (36-62)

Islands Region: 40 (33-48)^[[1]](#footnote-1)^

1. Manuel W Hetzel, Adnan A K Choudhury, and others, ‘Progress in Mosquito Net Coverage in Papua New Guinea’, *Malaria Journal*, 13 (2014), 242. [↑](#footnote-ref-1)
