# Supplementary material for: Repeated mosquito net distributions, improved treatment, and trends in malaria cases in sentinel health facilities in Papua New Guinea
Source: Malar J. 2019 Nov 12;18:364. doi: 10.1186/s12936-019-2993-6 (PMC6852945; doi:10.1186/s12936-019-2993-6)
Supplement: Supplementary file 8 — Additional file 8. Percentage of malaria cases treated with the previous first line treatment or partial treatment (mono-therapy), and with artemisinin-based combination therapy. [file 12936_2019_2993_MOESM8_ESM.docx]

# Additional file 8: Percentage of malaria cases treated with the previous first line treatment or partial treatment (mono-therapy), and with artemisinin-based combination therapy


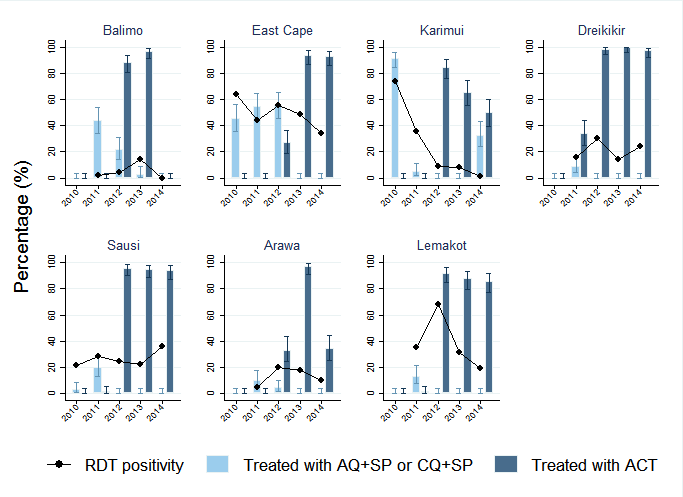


Annual malaria positivity and percentage of malaria cases treated with AQ+SP or CQ+SP, or with ACT.

AQ =amodiaquine, CQ = chloroquine, ACT = artemisinin-based combination therapy


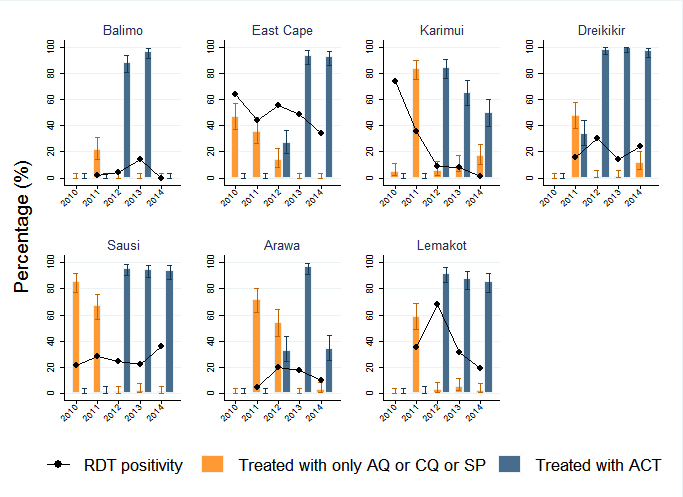


Annual malaria positivity and percentage of malaria cases treated with monotherapy (AQ or CQ or SP), or with ACT.

AQ =amodiaquine, CQ = chloroquine, ACT = artemisinin-based combination therapy
