# Supplementary material for: Repeated mosquito net distributions, improved treatment, and trends in malaria cases in sentinel health facilities in Papua New Guinea
Source: Malar J. 2019 Nov 12;18:364. doi: 10.1186/s12936-019-2993-6 (PMC6852945; doi:10.1186/s12936-019-2993-6)

# Additional file 9: Percentage of malaria cases treated with primaquine (for different RDT results), and with artemisinin-based combination therapy


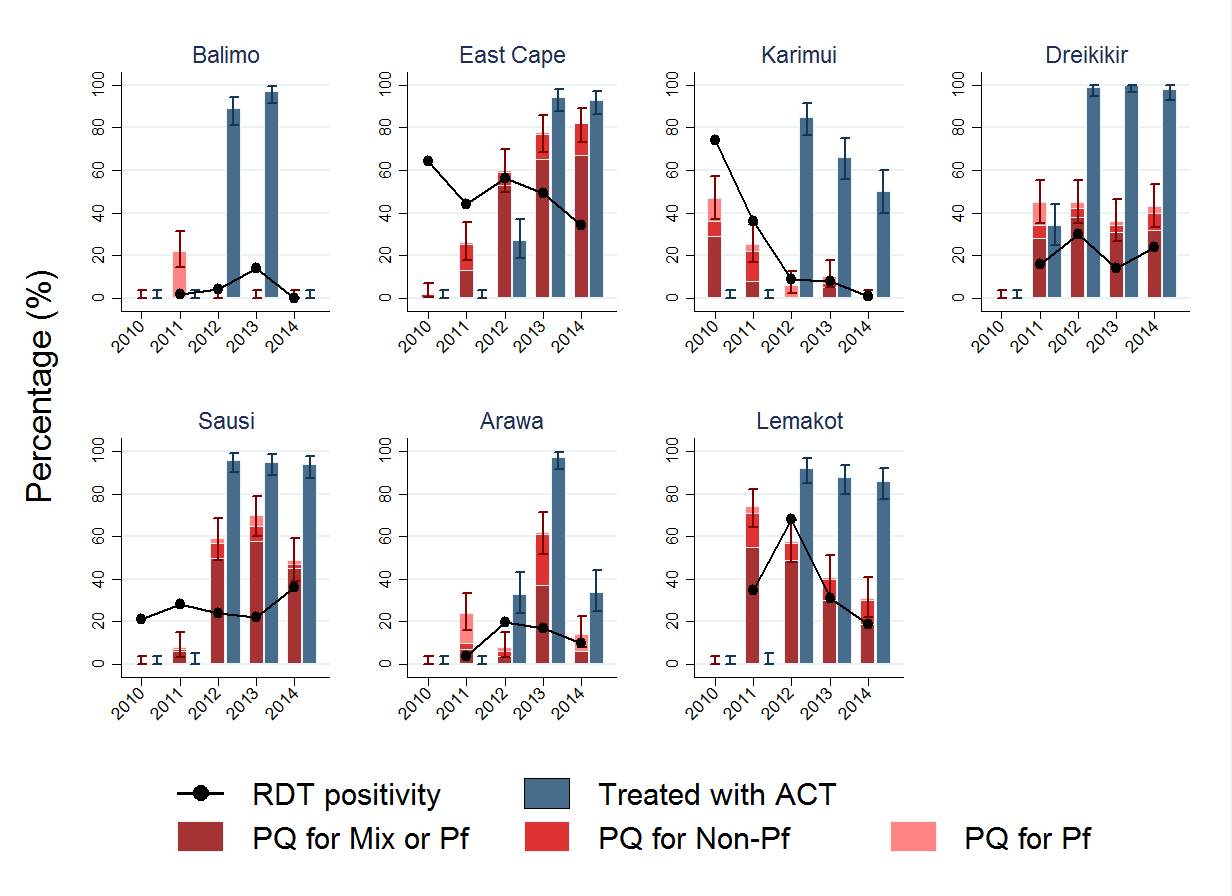

Supplement: Supplementary file 9 — Additional file 9. Percentage of malaria cases treated with primaquine (for different RDT results), and with artemisinin-based combination therapy. [file 12936_2019_2993_MOESM9_ESM.docx]
